# Accuracy of portrayal by standardized patients: Results from four OSCE stations conducted for high stakes examinations

**DOI:** 10.1186/1472-6920-14-97

**Published:** 2014-05-19

**Authors:** Lubna A Baig, Tanya N Beran, Andrea Vallevand, Zarrukh A Baig, Mauricio Monroy-Cuadros

**Affiliations:** 1 Institute of Public Health Jinnah Sindh Medical University Karachi, Karachi, Pakistan; 2 University of Calgary, 3330 Hospital Dr. NW, Calgary, AB T2N 4N1, Canada

**Keywords:** OSCE, Portrayal of SPs, Errors of assessments

## Abstract

**Background:**

The reliability in Objective Structured Clinical Exams (OSCEs) is based on variance introduced due to examiners, stations, items, standardized patients (SP), and the interaction of one or more of these items with the candidates. The impact of SPs on the reliability has not been well studied. Accordingly, the main purpose of the present study was to assess the accuracy of portrayal by standardized patients.

**Methods:**

Four stations from a ten station high-stakes OSCE were selected for video recording. Due to the large number of candidates to be evaluated, the OSCE was administered using four assessment tracks. Four SPs were trained for each case (n = 16). Two physician assessors were trained to assess the accuracy of SP portrayal using a station-specific instrument based on the station guidelines. For the items with disagreement a third physician was asked to review and the mode was used for analysis. Each instrument included case-specific items on verbal and physical portrayal using a 3-point rating scale (“yes”, “yes, but” and “not done”). The physician assessors also scored each SP on their overall performance based on a 5-item anchored global rating scale (“very poor”, “poor”, “ok”, “good”, and “very good”). SPs at location 1 were trained by one trainer and SPs at location 2 had another trainer. All SPs were employed in a high-stakes OSCE for at least the second time.

**Results:**

The reliability of rating scores ranged from Cronbach’s alpha of .40 to .74. Verbal portrayal by SPs did not significantly differ for most items; however, the facial expressions of the SPs differed significantly (p < .05). An emergency management station that depended heavily on SPs physical presentation and facial expressions differed between all four SPs trained for that station.

**Conclusions:**

Variation of trained SP portrayal of the same station across different tracks and at different times in OSCE may contribute substantial error to OSCE assessments. The training of SPs should be strengthened and constantly monitored during the exam to ensure that the examinees’ scores are a true reflection of their competency and devoid of exam errors.

## Background

The reliability in Objective Structured Clinical Exams (OSCEs) is based on variance introduced due to examiners, stations, items, standardized patients (SP), and the interaction of one or more of these sources of error [1]. The most important aspect of OSCEs is that it should measure the trait that it is intended to measure (i.e. validity) [2]. Hodges argued that validity studies in OSCEs do not capture the reality or authenticity of the assessment as the examination situation has a profound effect on the behaviour of the examinees and thus alters their behaviour accordingly [3].

Despite decades of improvement in the preparation of guidelines and curriculum for SPs [3,4], OSCEs that use SPs are subject to many measurement errors [1,5-7] including the inconsistency and inaccuracy of SP performance as well as their portrayal of the case. This latter form of error includes the degree of concordance between the SP’s appearance and symptom representation, adequacy of SP preparation, and appropriateness of the case [1,8-10].

Additional sources of SP error that have been identified include differences in portrayal of the same case by different SPs, demonstration of physical signs not related to the case, order and fatigue, and security breaches [11-17]. One threat to both the validity and reliability of OSCE scores is how well SPs portray the case [6,16].

Tamblyn et al. systematically evaluated SP accuracy for final year medical students from a Canadian and an American university [12,13]. They reported that SP portrayal was 93.4% accurate: history information was the most accurate (93.5%) and physical examination information was the least accurate (79.4%) [12-14]. Accuracy was worst at the beginning of day, improved by the 4^th^-6^th^ session and then deteriorated after the 7^th^-10^th^ session [13,14]. McKinley and Boulet assessed the effect of task sequencing on examinee performance and found no effect on OSCE scores. They, on the contrary, found that the scores improved as the examinees progressed through the OSCE stations, which, in their opinion, could have been due to increase confidence for attempting the exam18.

Sadeghi et al. studied portrayal of standardized patients for an eight station psychiatric OSCE held for residents. The examiners evaluated the performance of the standardized patients using a 5-point global rating scale (0 = very weak, to 4 = excellent). Their study found that in seven stations the examiners’ rating were identical (87.5%). None of the examiners rated standardized patients as “weak” or “very weak” - they were all rated at 2 or above (ok/fair) [11]. Their study suggested that the portrayal was accurate and appropriate for the cases.

Accuracy in SP portrayal of cases is critical for candidates attempting to demonstrate proficiency in clinical skills during the OSCE. The considerable time and resources required to evaluate SP accuracy might explain why few studies have addressed accuracy. This study extends the existing research on SP portrayal and critically assesses the portrayal of emotions, facial expressions and body language of SPs against the guidelines developed for the case.

The purpose of the present study was to assess the accuracy and realism of SP portrayal as rated by experienced clinicians. We critically reviewed the differences in portrayal across four tracks with SPs trained by the same trainer and for one case by two trainers across two locations. Background, history, physical and affect details documented within the four station-specific SP training booklets were considered while designing the assessment instrument and used by physician assessors for scoring the patient portrayal.

The Alberta International Medical Graduate (AIMG) Program was created by the Government of Alberta (Alberta Health and Wellness) in 2001 with a mission to increase the number of International Medical Graduates (IMGs) eligible to practice medicine in the province [18]. The AIMG Program uses, as part of its evaluation process, a 10-station OSCE to match qualified IMGs to allocated defined residency positions in Alberta. The successful candidates are then invited for a Multiple Mini Interview (MMI) with 9 stations of 9 minute each for assessing non-cognitive attributes of the IMGs. The scores on the MMI along with the OSCE results and candidates’ complete profile are then sent to the residency directors for matching to the defined residency positions in Alberta. Given that OSCEs typically exhibit some measurement errors [1,8,9] it is important to examine the sources of this error to increase accuracy of measurement.

## Methods

### Participants

A total of 142 IMGs participated in the OSCE. There were 68 (47.9%) females and 74 (52.1%) males, the graduation year ranged from 1982 to 2010, the youngest was 24 years of age and the oldest candidate was 55 years of age. One hundred and nine (109) candidates passed the OSCE and were invited to the Multiple Mini Interview (MMI).

### Physician assessors

Two family physicians assessed IMG performance. The first assessor was trained in Canada and has been an examiner for several Medical Council of Canada Exams and IMG OSCEs. He has been part of the IMG-OSCE committee for many years and developed OSCE stations for IMGs. The second assessor was trained outside of Canada, became qualified to practice through an IMG program and has been an examiner for IMG-OSCEs. The third physician, who reviewed selected tapes when there were differences in scoring between the two physicians, was also an IMG licensed in Alberta as a family physician.

### Procedure

Four stations from a ten-station high-stakes OSCE were selected for video recording at location 1 and one station (emergency management station) from the four selected at location 1 was video recorded at location 2 (refer to Table 1). Before entering the station, the examinees read the description of the case, which included 1-2 presenting complaints or the concerns of the patient. The SPs had been trained to present these complaints and additional information at the beginning of the interaction as part of the “opening statement”. The script of the SP also included details on the responses that they had to give only if asked for by the examinee.

**Table 1:** Cases included in the study

**Case**	**Sex**	**Age range in years**	**Complaints**	**Tasks**	**Diagnosis**
A	Male	40-50	Chest pain	History and management	Gastroesophageal reflux disease (GERD)
B	Female	23-28	Acute abdomen	History, physical examination and management	Ectopic Pregnancy
C	Female	40-45	Concerned about memory loss in father	History and counselling	Alzheimer
D	Female	13-18	Dysmenorrhea Requesting oral contraceptives	History and counselling	Counselling a teenager on contraception

The cases were selected after discussion with the research team and included assessment of varied skills (counseling, physical examination, history taking, and management of emergencies). The other major reason for selecting them was that they were heavily dependent on facial expressions, tone of voice, and demonstration of fatigue by the SPs by the end of encounter. Due to the large numbers of candidates, the OSCE was conducted using four tracks (which were labelled: Track 1, Track 2, Track 3, Track 4) and two sessions (morning and afternoon), which required four SPs be trained for each station.

Six SP-candidate interactions were selected from each assessment track – three interactions from the morning session (first, middle, and last) and three from the afternoon session (first, middle, and last). This was in keeping with the literature that suggests that there could be an effect of sequencing on the scores of the participants [12,14,19]. In this study we did not look at the effect on candidates as our main purpose was assessing accuracy of portrayal by SPs across all tracks.

The physician assessors reviewed the SP guidelines developed for case portrayal and the checklist used by the examiners for scoring the candidates. They viewed the video recordings and scored SP portrayal on 6 SP-candidate interactions within each track (four tracks times’ four cases times six candidates). The physician assessors were trained for each station using videos that were not included in the study and practiced with the checklist developed for assessing the SP portrayal.

### Instrument

The checklists for each of the four cases included specific items on verbal and physical portrayal rated on a 3-point scale (“yes”, “yes, but” and “not done”), for example:

•SP effectively portrayed his concerns at the opening statement

•SP’s tone of voice was anxious as he is worried about a heart attack.

•SP portrays screams of pain when deep palpation is released

•SP effectively discussed her concerns about her mother finding out that she is in a relationship.

The physician assessors also scored the SP on the overall performance using a 5 item anchored global rating scale (“very poor”, “poor”, “ok”, “good”, and “very good”). The SP-candidate interactions used for training the physician were not included in the study.

### Analyses

Inter-rater consistency was calculated using Cohen’s Kappa, which ranged from 0.80 to 0.89 for all the four cases across the two physicians; the third physician only reviewed selected cases with disagreement. For items with disagreement, the modal value between the three assessors was used for analysis. Internal consistency of scores given by both physician raters was calculated by Cronbach’s Alpha. Chi-square was used to assess significant differences in the SPs’ portrayal of guidelines developed for the case.

## Results

There was 85% agreement between the two physician assessors. There was full agreement on verbal portrayal and facial expressions across all cases with the only disagreement on cases where the videos were not clear (sound and/or picture) or the SP was not directly in front of the camera. There was no effect of time on portrayal for all the cases irrespective of the track and the location (Case B was the only station recorded at two locations).

### Case A

This was a history and management case and the management response of the candidates depended heavily on the SP’s history and his facial expressions showing concern for his current health status (chest pain). The internal consistency of scores, calculated using Cronbach’s alpha, was 0.744. There was significant difference in portrayal across tracks for the opening statement, facial expressions and for asking questions from the candidate at 9 minutes (refer to Table 2). In all the tracks, the SPs did not give information without being asked for questions on cardiac risk factors, past history, and on diet and weight. In one track the SP gave information once without being asked for questions on present history. There were significant differences across tracks for SPs’ overall portrayal (p < 0.01), verbal (p < 0.05), and facial expressions (p < 0.05). On combining the total items (n = 36, last row on Table 2) with accurate portrayal, there were significant differences across all tracks for comparison between “yes, yes/but and not done” (p <0.001).

**Table 2:** Case A - SP portrayal of history and management

**Questions**	**Track 1**		**Track 2**		**Track 3**		**Track 4**		**P**
	**N = 6**		**N = 6**		**N = 6**		**N = 6**		
	**Yes**	**Yes/but**	**Yes**	**Yes/but**	**Yes**	**Yes/but**	**Yes**	**Yes/but**	
SP effectively portrayed his concerns at the opening statement	2	4	6	0	0	6	4	2	0.004
SP’s tone of voice was anxious as he is worried about a heart attack	0	6	0	6	2	4	3	3	0.078
SP’s facial expressions were appropriate for questions on present history	6	0	6	0	3*	0	6	0	0.016
SP gave information without asking for questions on history	0	0	0	0	0	0	1**	0	0.372
SP had appropriate expressions while asking questions at 9 minutes	1	5	2	3	1	2*	4	1	0.046
SP’s tone of voice was appropriate while asking questions at 9 minutes	0	6	0	5	1	5	4	1	0.029
Accuracy (6 items X 6 cases = 36)	6	21	18	14	5	27	22	7	0.000
	17%	58%	50%	39%	14%	75%	61%	19%	
Overall (yes plus yes/but)	Overall = 75%		Overall = 89%		Overall = 89%		Overall = 81%		

*Could not observe the remaining cases for the SP due to direction of the camera.

**The SP gave out the information without being asked only once.

### Case B

This was an emergency management case and the SP was trained to portray distended stomach during the examination. There were significant differences in portrayal for physical appearances and facial expressions. The internal consistency measured by Cronbach’s alpha was 0.40. This was the only case that was videotaped at two locations and had different SP trainers for both locations. The additional purpose for this station was to investigate whether there were differences in training by the two trainers based on the same guidelines for SPs across two locations for a case that depended heavily on physical portrayal and facial expressions. The SPs were asked to look lethargic at the end of the case. We found a significant difference across two locations (p < 0.05); however within each location the SPs mostly did not portray being lethargic at the end (refer to Table 3). There were significant differences (p < 0.001) between the two locations for SPs portraying distended abdomen. The SPs were instructed not to react to additional physical assessment not related to the case and, except for one SP who reacted only once, none of the SPs at any location reacted. The SPs also did not give out information without being asked for questions on social history and on other systems of the body. There were significant differences between SPs across all tracks and both locations for questions on presenting complaints and gynaecological/obstetrical history (Table 3). There were significant differences across tracks and across locations for the SPs’ overall portrayal (p < 0.05), verbal (p < 0.05), and facial expressions (p < 0.05). There were no significant differences across tracks and across locations for SP portrayal of lethargy at the end of the case for each candidate. On combining the total items (n = 60, last row on Table 3) with accurate portrayal there were significant differences across all tracks for comparison between “yes, yes/but and not done” at location one (p-value <0.05), location two (p-value <0.05), and for both location together (p-value <0.04).

**Table 3:** Case B - portrayal of case by SPs across all tracks and both locations

	**Location 1**								**Location 2**			
	**Track 1**		**Track 2**		**Track 3**		**Track 4**		**Track 1**		**Track 2**	
	**N = 6**		**N = 6**		**N = 6**		**N = 6**		**N = 6**		**N = 6**	
**Questions**	**Yes**	**But**	**Yes**	**But**	**Yes**	**But**	**Yes**	**But**	**Yes**	**But**	**Yes**	**But**
SP effectively portrayed being alert at beginning of case	6	0	6	0	6	0	5	1	6	0	6	0
SP’s face looked pale^♦♣^	0	2**	0	0	0	0*	3	3	3	0*	6	0
SP’s mouth looked dry and she either asked for water or said that she had been drinking frequently	2**	0	6	0	2	4	0	5*	3	3	3	3
SP’s tone of voice was appropriate for presenting the chief complaint	6	0	6	0	6	0	6	0	6	0	6	0
SP’s facial expressions were appropriate for responding to the questions on history^♣♦^	6	0	6	0	0	0**	1	4*	1	1*	6	0
SP gave information without asking for questions on presenting complaints*^♣^	0	0	0	0	6	0	0	0	0	0	0	0
SP gave information without asking for questions on gynaecological/obstetrics history^♣♦^	1	0*	5	0*	6	0*	3	3	1	1*	4	1*
SP portrays screams in pain when deep palpation is released	4	0*	5	0	6	0	2	1**	4	1**	2	0**
SP reacted on additional physical assessment not related to the case	0	0	0	0	0	1	0	0	0	0	0	0
On inspection the abdomen seemed distended^♣^	0	0**	0	0**	6	0	0	1**	0	0**	0	0*
Accuracy***	25	2	34	0	38	5	20	18	18	6	27	4
	42%	3%	57%	05	63%	8%	33%	30%	30%	10%	45%	6%
(10 items X 6 cases = 60)												
Overall yes and yes/but	45%		57%		72%		63%		40%		51%	

*Could not observe the remaining cases for the SP.

**SP did not portray appropriately the remaining cases.

^♣^P-value for Location 1 < 0.05.

^♦^P-value for Location 2 < 0.05.

***P-value for comparison of all tracks for both Locations <0.04.

### Case C

This was a counselling case where the daughter was concerned about her father’s memory loss. The case depended heavily on the SPs’ facial expressions and history. The internal consistency reliability of the portrayal scores was 0.41 (Cronbach’s alpha). Overall the SPs in all tracks portrayed the case appropriately although there were significant differences in facial expressions especially for one track (Table 4). The SPs occasionally gave out information on the father’s recent memory loss, distant memory, general health, depression and confusion to candidates without being asked. However, the SPs did not volunteer information without being asked for questions on family and social background in any track. The overall portrayal only differed significantly for facial expressions across tracks (p < 0.001) with no significance across tracks for verbal and overall portrayal. On combining the total items (n = 54, last row on Table 4) with accurate portrayal there were significant differences across all tracks for comparison between “yes and not done” (p <0.04).

**Table 4:** Case C - portrayal of case across all tracks

**Questions**	**Track 1**		**Track 2**		**Track 3**		**Track 4**		**P-value**
	**N = 6**		**N = 6**		**N = 6**		**N = 6**		
	**Yes**	**Yes/but**	**Yes**	**Yes/but**	**Yes**	**Yes/but**	**Yes**	**Yes/but**	
SP’s tone of voice was appropriate for the opening statement	6	0	6	0	6	0	6	0	1.0
SP seemed worried but composed	6	0	6	0	6	0	6	0	1.0
The SP’s facial expressions were appropriate for responding to the questions on fathers memory	6	0	0	0*	6	0	5	0*	0.0000
SP cried once or twice during silence	6	0	6	0	6	0	6	0	1.00
SP gave information without asking for questions on father’s recent memory loss^♣^	2	0**	1	0**	0	0**	0	0**	0.242
SP gave information without asking for questions on father’s distant memory^♣^	1	0**	0	0**	0	0**	0	0**	0.372
SP gave information without asking for questions on general health issues of her father^♣^	0	0**	0	0**	0	0**	1	0**	0.372
SP gave information without asking for questions on depression and mood changes^♣^	0	0**	0	0**	0	0**	1	0**	0.372
SP gave information without asking for questions on confusion/orientation in time and place^♣^	1	0**	0	0**	2	0**	0	0**	0.242
Accuracy (9 items X 6 cases = 54)	28	0	19	0	26	0	25	0	0.04***
	58%	0%	40%	0	54%	0%	52%	0%	
Overall (yes plus yes/but)	58%		40%		54%		52%		

*Could not observe the SP due to direction of the camera.

**SP did not give out the information without asking.

^♣^The SPs gave out the information once.

***The comparison was between accurate portrayal and not done as there were 0 values for all “overall yes/but”.

### Case D

This was a case of a teenage girl requesting oral contraceptive pills and complaining of menstrual irregularities. The internal consistency reliability of scores of accuracy of portrayal was 0.56,. There were significant differences in the SPs’ portrayal across tracks on discussing concerns about her mother finding out that she is in a relationship (Table 5). There were significant differences across tracks for the SPs’ overall portrayal (p < 0.05), verbal (p < 0.05), and facial expressions (p < 0.05). The SPs across all tracks were consistent in not giving out information without being asked on past medical history, sexual history, medications, smoking and alcohol consumption. On combining the total items (n = 42, last row on Table 5) with accurate portrayal there were significant differences across all tracks for comparison between “yes and not done” (p-value <0.04).

**Table 5:** Case D - portrayal of case across all tracks

**Questions**	**Track 1**		**Track 2**		**Track 3**		**Track 4**		**P-value**
	**N = 6**		**N = 6**		**N = 6**		**N = 6**		
	**Yes**	**Yes/but**	**Yes**	**Yes/but**	**Yes**	**Yes/but**	**Yes**	**Yes/but**	
SP effectively portrayed the concerns regarding her menstrual cycle	4	0	6	0	6	0	6	0	1.0
SP’s tone of voice was appropriate for a young girl wishing to get pills	6	0	6	0	6	0	6	0	1.0
SP effectively discussed her concerns about her mother finding out that she is in a relationship	1	2*	2	2*	0	0*	0	0*	0.046
SP’s facial expressions were appropriate for responding to the questions family and social relationships	1	2*	2	2*	0	0*	0	0*	0.687
SP effectively portrayed her relationship with the boy friend	1	2*	2	2*	0	0*	0	0*	0.538
SP had appropriate expressions while asking for pills at 7 minutes	3	1	6	0	6	0	5	0*	0.283
SP’s tone of voice was appropriate when asking about the confidentiality of her discussion with the physician	4	0	3	1*	6	0	5	0*	0.328
Accuracy (7 items X 6 cases = 42)	20	7	27	7	24	0	22	0	0.04**
Overall (yes plus yes/but)	48%		64%		57%	0	52%	0%	
	17%		17%		57%		52%		
	64%		81%						

*SP did not portray appropriately.

**The comparison was between accurate portrayal and not done as there were 0 values for all “overall yes/but”.

## Discussion

The key findings of the study were that the scores for assessing SP portrayal demonstrated good internal consistency reliability for Cases A and D. Meanwhile, Cases B and C had a low Cronbach’s alpha (0.40), which in our opinion, is likley related to videotaping deficiencies (e.g., camera angles). In Case C, the low reliability could be due to the physician assessors not getting an adequate view of the SPs while rating, which lead to inconsistency in scores. Case B, the emergency case, was heavily dependent on physical portrayal, with gradual change in portrayal (e,g., lethargy) during the examinee and SP encounter. The physicians assessors, due to the positioning of the camera, were not able to judge consistently if the SPs’ were portraying these affect changes accurately, or not.

The SPs trained by the same trainer mainly differed in facial expressions across all tracks. The verbal portrayal by SPs did not significantly differ for most items and the facial expressions of the SPs differed significantly across all tracks. The emergency management case that depended heavily on the SPs’ physical and facial expressions differed across all tracks and both locations. There was no major difference in portrayal at different time points during the OSCE hence we may say with some confidence that candidates scores were likely not affected by SP portrayal.

The differences in accuracy of portrayal varied across stations and tracks (different SPs portraying the same case). The highest range of variation among SPs was in the case (Case D) of a teenage girl requesting contraceptives (52% to 81% accurate portrayal). The reason could be either that the SP was a young teenage girl and/or that the portrayal depended heavily on facial expressions and history. The other counselling case which depended on facial expressions and history also had a wide range of accuracy across tracks (42% to 62% accurate portrayal) and overall accuracy of portrayal by the SPs for that case was lowest.

The accuracy of portrayal ranged from 42% to 89%, with the highest for the history and management case (75% to 89%) and lowest was for the counselling case (42% to 62%). These values are lower than the Tamblyn study which found 93% accuracy for portrayal [12-14]. We did not find any major difference in portrayal over time within each SP, unlike the previous studies [14,15,20]. Like the Tamblyn study we also found that the major differences in presentation were related to physical portrayal [12-14].

There was no major difference in portrayal at different time points during the OSCE. This result is inconsistent with the Tamblyn [14] study where they found that accuracy was worst at the beginning and end and accurate during the middle of the day. The McKinley and Boulet [19] study found no effect on the sequencing of OSCE stations, which may also reflect that the SP portrayal over time did not change. On the other hand, the score drift is an indication that some of the error may be attributed to SP portrayal with different administration of the OSCE stations [21]. As we did not look at the examinees scores over multiple administrations we cannot comment on the score drift, however we can say with some confidence that it does not appear that candidate scores were affected by SP portrayal [19,21].

Overall the present findings are in concordance with the Sadeghi et al. study as overall rating of portrayal was “ok” or more for any SP (refer to global rating scale in the methods section) [11]. In the present study the SPs provided a good portrayal of the case; however we found differences across tracks and for one case across two locations.

We argue that despite concerted efforts by medical educationists, SP training is still not close to the real doctor-patient encounters. The SPs have formed an association with regular meetings in the western world and should now join hands with the medical educationists and enhance the training and skills of SPs. The SP trainers and OSCE organisers/mangers should regularly evaluate the accuracy of portrayal for quality enhancement. The checklists we utilized could be further validated using larger samples and the errors in scores should also be evaluated using larger samples. Even though the portrayal was not as accurate as desired, the SP based examination is still the best form of clinical skills assessment [1,4-6].

## Conclusions

The results of the present study indicate that focus on the emotions, facial expressions and body language of SPs during training should be emphasized. We should continue to strengthen and ensure the standardization of training especially if more than one SP is used for each case and also if there is more than one track during OSCE. At this time SP portrayal is not regularly assessed, and it is generally assumed that they are following guidelines and that training is appropriate. It is our suggestion that in SP based examinations the assessors give a formal or informal feedback to the SPs and their trainers after the exams. The instrument that we developed had good reliability of scores given by the physician assessors and can be used with modifications for most SP-based exams. Improved training of SPs can improve the quality of SP-based exams. The major differences were mainly for physical portrayals or facial expressions; henceforth, portrayal can be improved with better training of SPs and preferably use of actors for exams. The instruments developed for assessing portrayal by physician assessors should be tested with larger data for adducing evidence of validity.

### Limitations

This study is from a small sample of IMGs aspiring for licensure in Canada. As the results are based on video recordings, any defect in recording (clarity of sound or picture) may have caused an error in scoring by the physician assessors. As we did not get approval to use the candidates’ scores, we could not do a generalizability analysis to look at the sources of error and score drift. The major limitation of this study was due to inaccessibility of examinees scores for assessing whether portrayal across tracks was a source of error, or not.

### Ethical approval

Ethical approval was received from the University of Calgary prior to the study. The SPs and candidates signed a consent form giving approval for video recording. During data entry and analysis the candidates and SPs were given different code numbers which could not be traced to their personal identification to ensure confidentiality and privacy.

## Competing interests

We declare that none of the authors had any competing interests for the conduct or publication of this study. The publication of this study will not cause any gain or harm (financial or any other kind) to any organization associated with this study.

## Authors’ contribution

LAB carried out the research with help from TNB, AV and MMZ. ZAB was the research assistant for the project and collected and analysed the data. LAB wrote the first draft, ZAB did the literature review, TNB and AV reviewed and suggested modifications to the first draft. The reviews and edits were done in the same order sequentially as listed in the authorship of the manuscript. All authors read and approved the final manuscript.

## Pre-publication history

The pre-publication history for this paper can be accessed here:

http://www.biomedcentral.com/1472-6920/14/97/prepub
